# Supplementary material for: Impact of grain boundaries on efficiency and stability of organic-inorganic trihalide perovskites
Source: Nat Commun. 2017 Dec 20;8:2230. doi: 10.1038/s41467-017-02331-4 (PMC5738431; doi:10.1038/s41467-017-02331-4)
Supplement: Supplementary file 1 — Supplementary Information [file 41467_2017_2331_MOESM1_ESM.pdf]

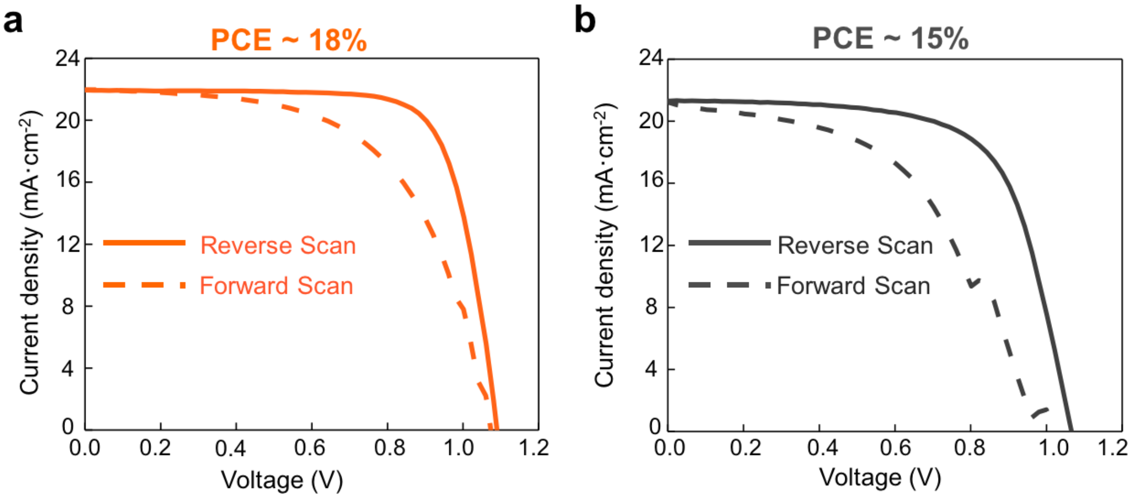

c

		J_{sc}(mA·cm^{-2})	V_{sc}(V)	FF	PCE (%)
~15%	Reverse	21.33	1.066	0.667	15.18
	Forward	21.19	1.018	0.487	10.51
~18%	Reverse	21.94	1.089	0.755	18.06
	Forward	21.98	1.073	0.585	13.79

Supplementary Figure 1. Standard AM1.5 solar cell device performance. I-V characteristics of the solar cell device made from the $MAPbI_3$ thin films of (a) PCE ~ 18% and (b) PCE ~ 15% used in this work under the standard AM1.5 illumination. Both forward and reverse curves are shown. The scan speed was 0.2 V·s^{-1}. (c) Table of the device parameters, listing the short-circuit current density J_{sc}, open-circuit voltage V_{oc}, fill factor (FF), and PCE of both films.

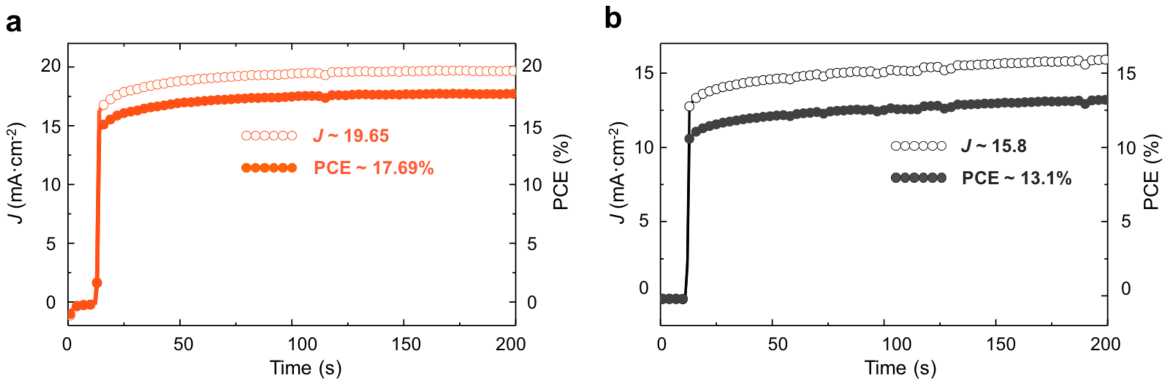

Supplementary Figure 2. Stable power output (SPO) near maximum power point. The SPOs are about (a) 17.69% and (b) 13.1% for cells based on large and small grains, respectively. These values are between the reverse and forward scans results but closer to the reverse scan results in Supplementary Figure 1.

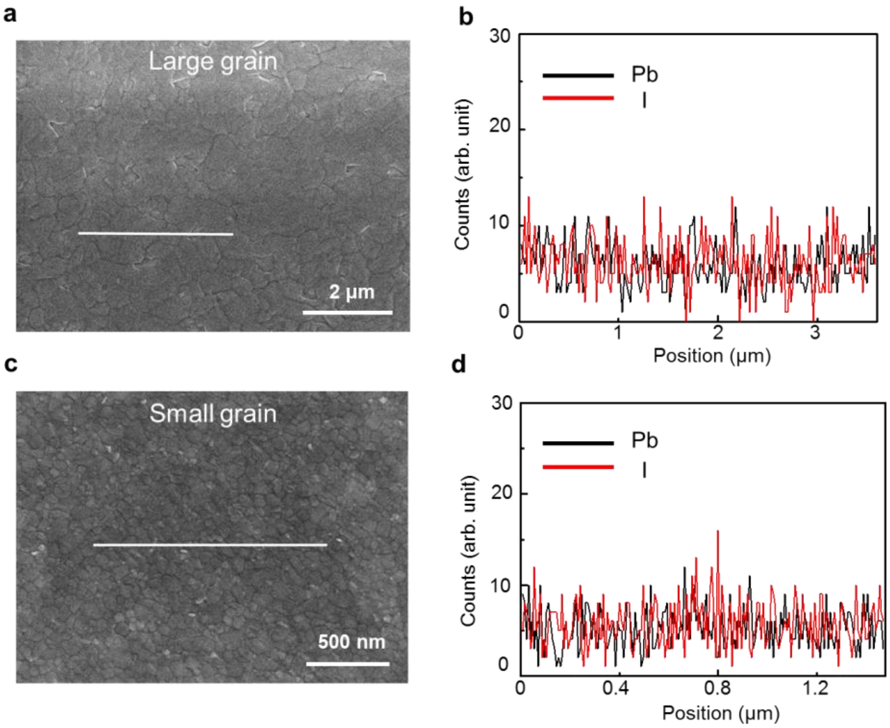

Supplementary Figure 3. Energy-dispersive x-ray spectroscopy (EDS) results. (a) EDS images of the large grain and (b) small grain samples. (c) and (d) show the corresponding line scan results. There is no clear correlation between the Pb and I distribution and the grain morphology, e.g., grain versus grain boundary. For both samples, Pb and I stay roughly constant across multiple grains within experimental noise. The EDS analysis of the entire sampling area shows that the ratio of I/Pb is about 3.0±0.3 for the small grain sample and 2.9±0.3 for the large grain sample. The uncertainty is caused by the EDS detection limit (about 0.5 to 1%). Thus, it is reasonable to conclude that these two samples have essentially the same composition within experimental error and no local variations are observed across multiple grains.

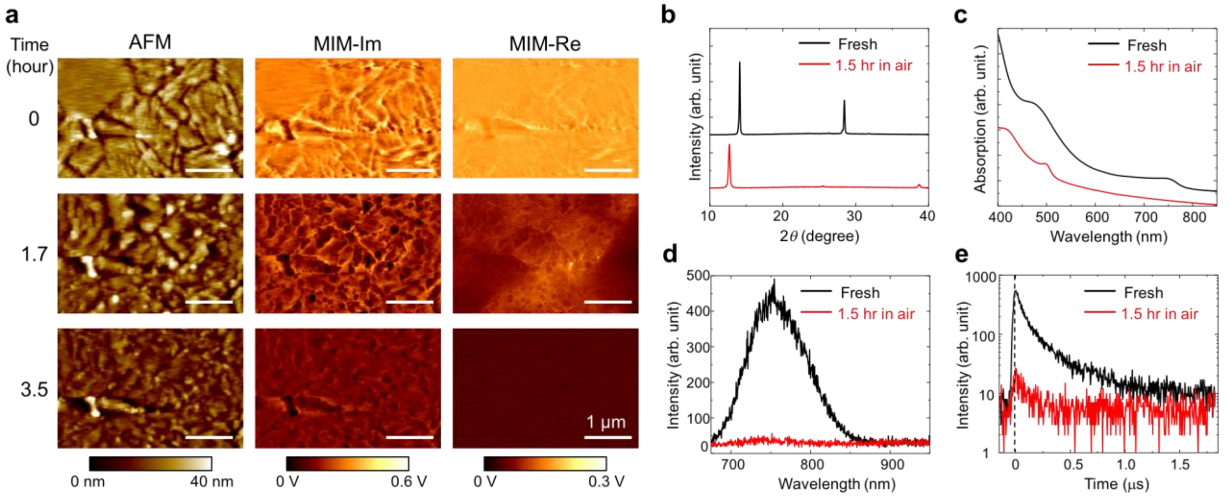

Supplementary Figure 4. Degradation process of uncapped perovskite thin films. (a) AFM and MIM images of a bare perovskite thin film exposed to the ambient environment, showing the rapid degradation over several hours. All scale bars are 1 μm. (b) Comparison of the X-ray diffraction pattern, (c) optical absorption, (d) photoluminescence (PL) spectrum, and (e) time-resolved photoluminescence (TRPL) data of a bare $MAPbI_3$ thin film between $t = 0$ (fresh) and $t = 1.5$ hours in air. The film is degraded after the 1.5-hour exposure, consistent with the MIM data.

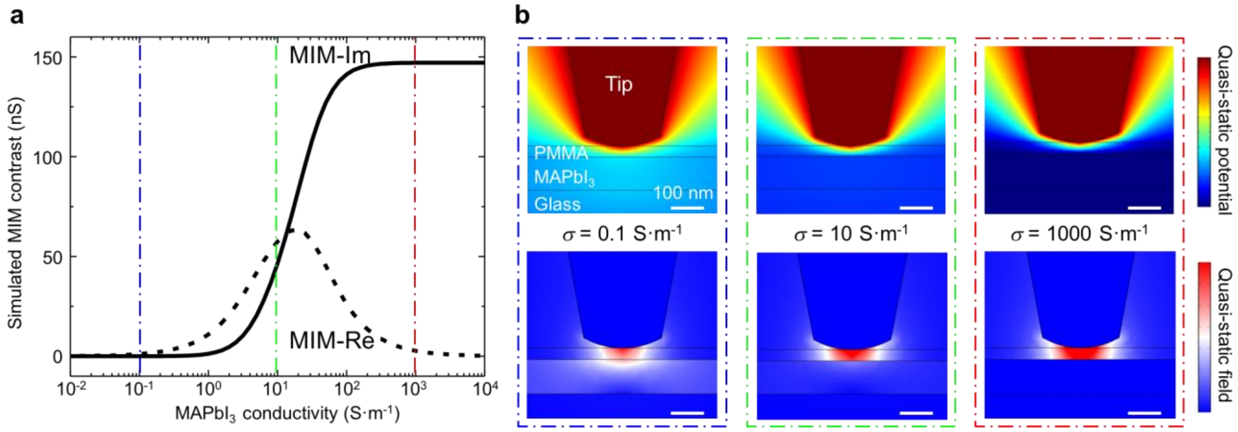

Supplementary Figure 5. 2D FEA simulation of the $MAPbI_3$ thin film (a) Simulated MIM contrast (with respect to zero conductivity) as a function of the conductivity σ of the $MAPbI_3$ layer. The three characteristic σ's color-coded in (b) are labeled by dash-dotted lines. (b) Quasi-static potential (upper panels) and displacement field (lower panels) distributions at $\sigma = 0.1\ S\cdot m^{-1}$ (left), 10 $S\cdot m^{-1}$ (middle), and 1000 $S\cdot m^{-1}$ (right). All scale bars are 100 nm.

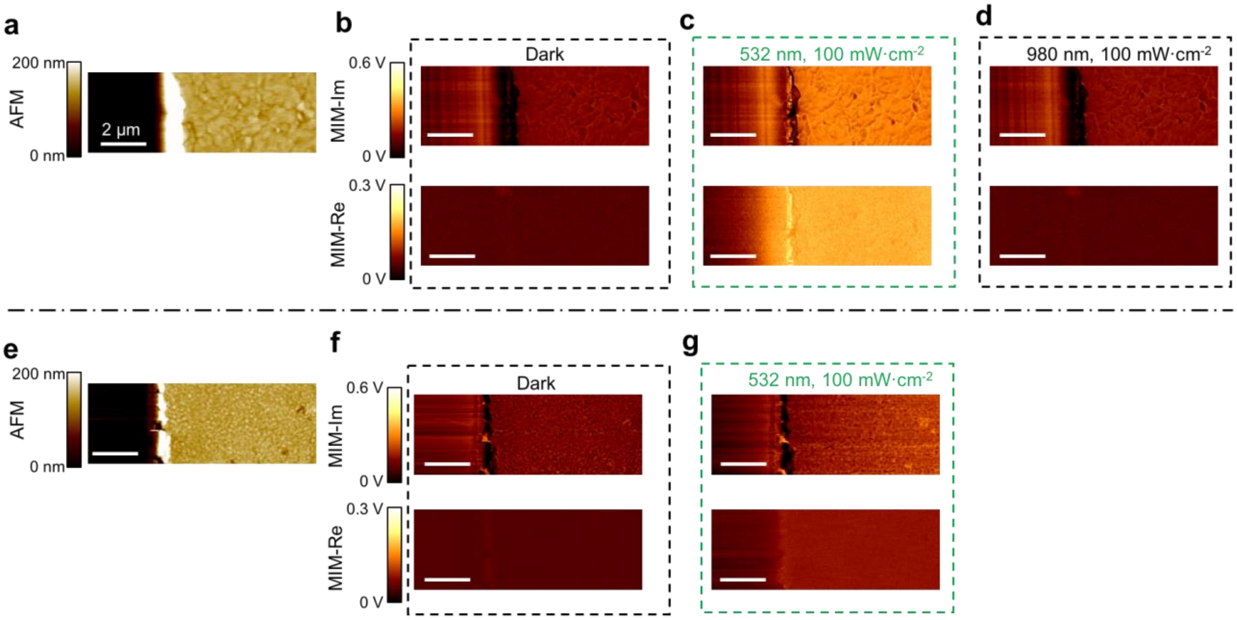

Supplementary Figure 6. AFM and MIM images of $MAPbI_3$ thin films. (a-d) AFM and MIM images of the 18% and (e-g) 15% PCE samples under different illuminating environment, with (a) and (e) showing the AFM topography, (b) and (f) showing MIM-Im and MIM-Re images with no laser illumination, (c) and (g) showing MIM images under 532-nm laser illumination of 100 mW·cm^{-2} power density, and (d) showing the MIM images under 980-nm laser illumination of 100 mW·cm^{-2}.

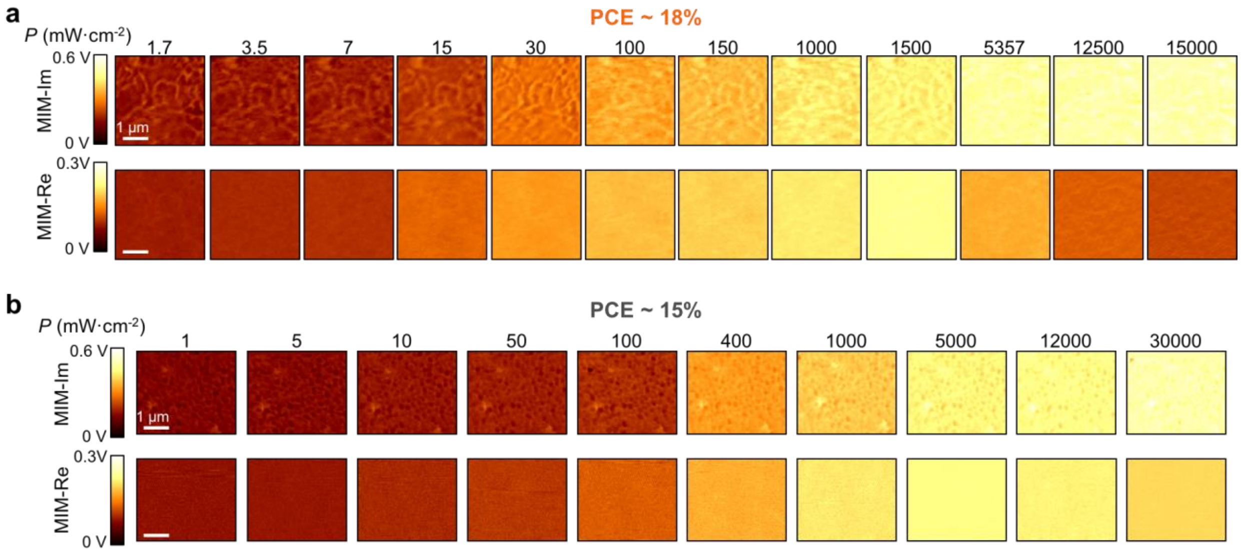

Figure S7. Complete set of MIM data. (a) MIM data of the 18% and (b) 15% PCE samples as a function of incident 532-nm laser power density. The averaged signals in these images are plotted in Figures 3a and 3b. All scale bars are 1 μm.

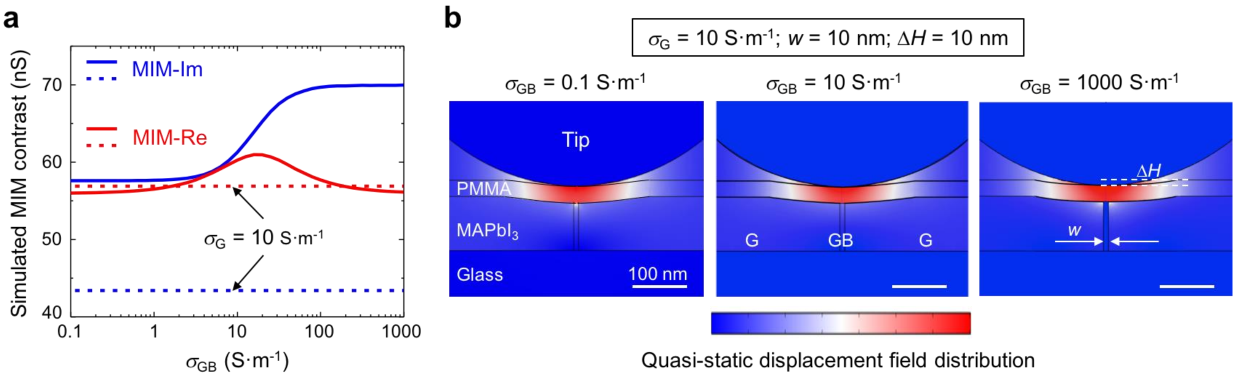

Supplementary Figure 8. 3D FEA simulation of grain boundaries on the $MAPbI_3$ thin film. (a) 3D simulation as a function of the conductivity of the grain boundary (GB) σ_{GB}. The MIM signals when the tip locates on top of the grains with $\sigma_G = 10\ S\cdot m^{-1}$ are also labeled in the plot as dashed lines. (b) Quasi-static displacement field distribution around the tip-sample junction at σ_{GB} = 0.1 (left), 10 (middle), and 1000 $S\cdot m^{-1}$ (right). All scale bars are 100 nm.

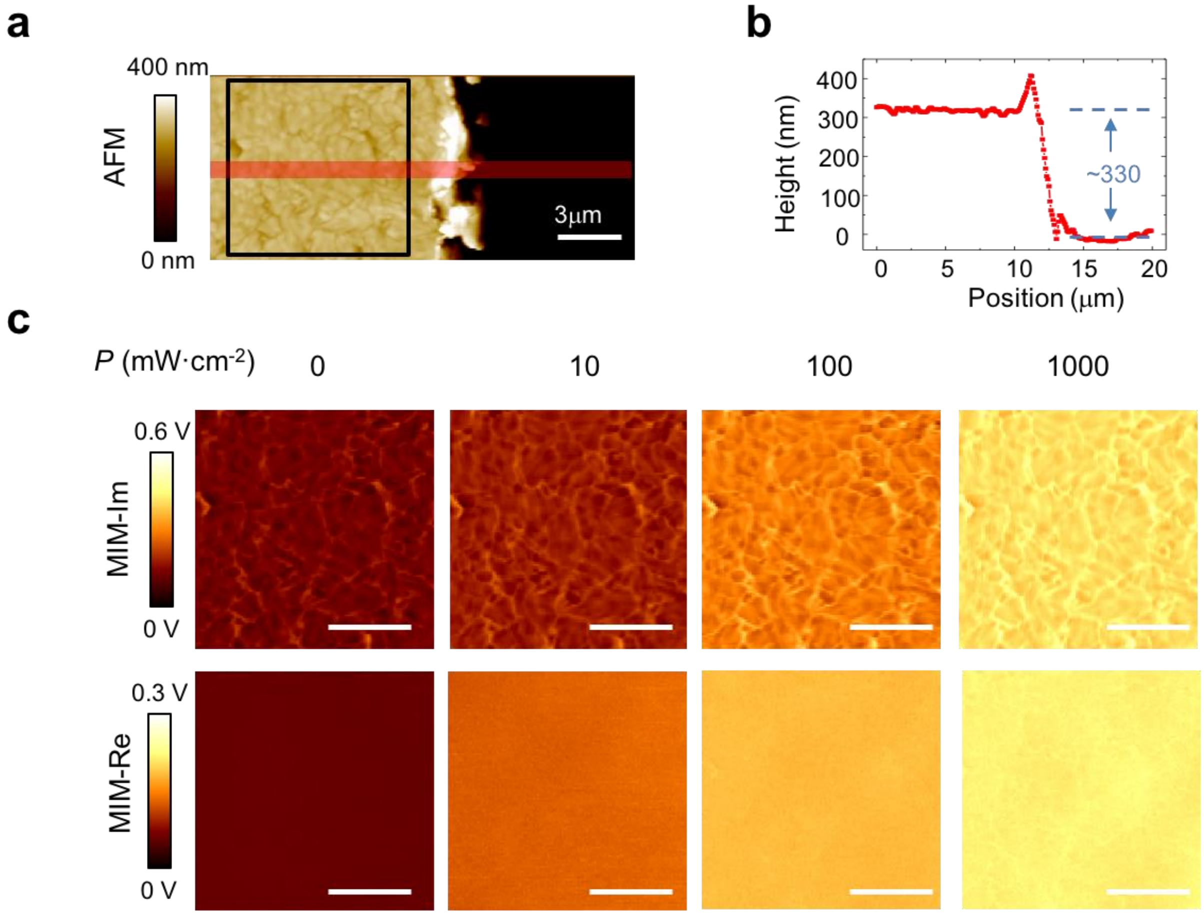

Supplementary Figure 9. AFM and MIM images of a thick $MAPbI_3$ film. (a) AFM topography of the 300-nm film made from the 18% PCE sample. (b) Line profile in (a), showing a thickness of about 330 nm (300 nm $MAPbI_3$ and 30 nm PMMA coating) above the substrate. (c) MIM images within the solid square in (a) as a function of the power density. All scale bars are 3 μm.

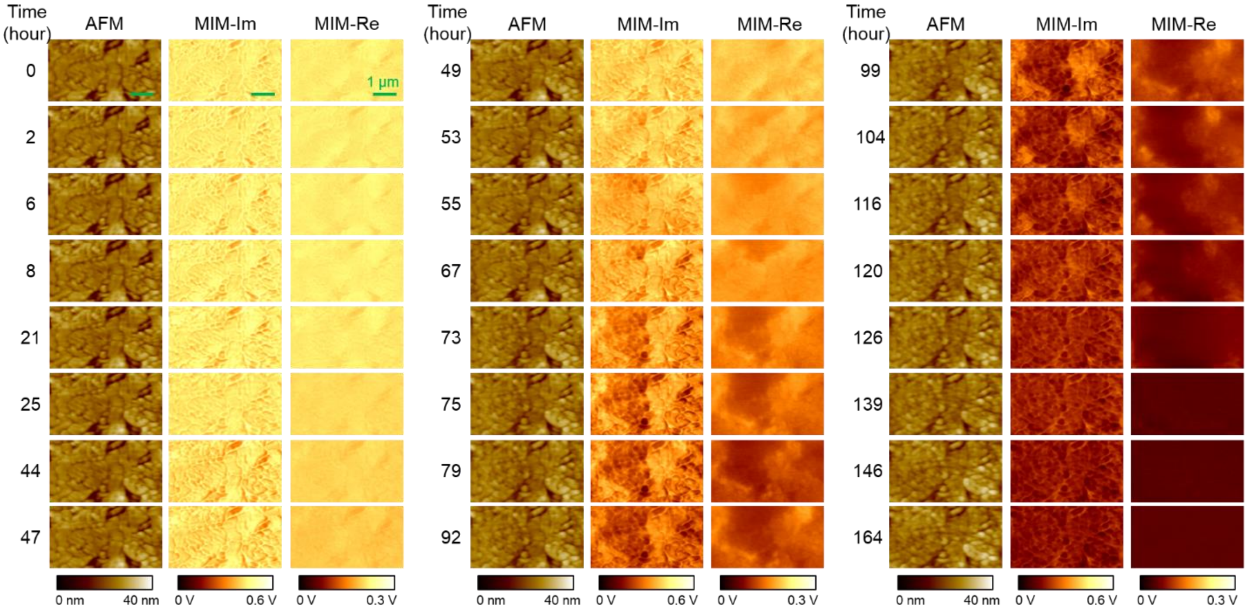

Supplementary Figure 10. Complete set of the time dependence data. The spatially resolved degradation process of the PMMA/$MAPbI_3$ thin film is shown over the period of one week. All images are 4.8 μm × 2.6 μm in size and the scale bars are 1 μm. The laser power is set to be 1000 $mW{\cdot}cm^{-2}$ for this study. The sample is only illuminated during the MIM imaging, about 30 min for each frame. The total amount of illumination time is thus around 9 hours for this experiment.

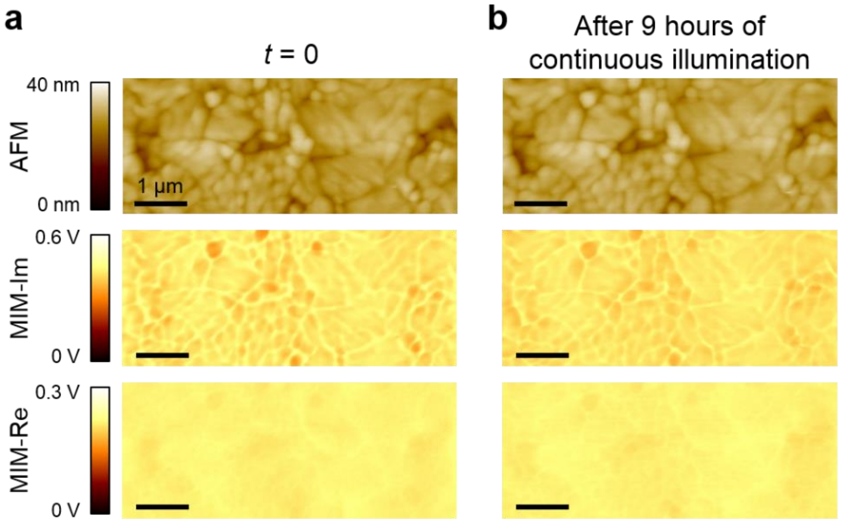

Supplementary Figure 11. Control experiment with continuous laser illumination. (a) AFM and MIM images at t = 0 and (b) after 9 hours of continuous illumination by the 532nm laser and power intensity 1000 mW·cm^{-2}. All scale bars are 1 μm.

Supplementary Note 1. In order to explore the possible effect of surface recombination on the MIM imaging, we have performed additional control experiments on a thicker $MAPbI_3$ film made from the 18% PCE sample. The film thickness of 300 nm is evident from the AFM data in Supplementary Figure 9a and the line profile in Supplementary Figure 9b. Note that the thickness is comparable to the solar cell devices (350 nm) that demonstrate a PCE of 18% in Supplementary Figure 1. The MIM data in Supplementary Figure 9c are very similar to those acquired on the 100 nm film (Supplementary Figure 7). In particular, the MIM-Re images, which are less affected by the topographic crosstalk than the MIM-Im images, are again uniform across grains and grain boundaries. The results suggest that the MIM signals on both the thin (100 nm) and thick (300 nm) films in our study are not dominated by the surface recombination effect. However, we also acknowledge that surface defects are generally present in $MAPbI_3$ devices and their effects cannot be totally excluded in our measurements.

Supplementary Note 2. As a control experiment, we have also studied the effect of continuous laser shining (532 nm, 1000 $mW \cdot cm^{-2}$) for 9 hours, equivalent to the total amount of illumination in Supplementary Figure 10. As shown in Supplementary Figure 11, no change is observed between $t = 0$ and $t = 9$ hr. We thus conclude that the illumination by itself is insufficient to cause the drastic suppression of photoconductivity in the degradation process discussed in Figure 5 and Supplementary Figure 7.
